# Metropolitan Hospital Sunday Fund

**Published:** 1906-03-24

**Authors:** 


### METROPOLITAN HOSPITAL SUNDAY FUND.

#### THE FUND TO BE INCORPORATED.

A Special general meeting of the constituents of the Metropolitan Hospital Sunday Fund was held at the Mansion House on Tuesday last (20th instant), the Lord Mayor (Alderman Vaughan Morgan), the President and Treasurer, in the chair.

The Rev. Canon Fleming, Vicar of St. Michael's, Chester Square, moved the following resolution recommended by the Council, namely :—

> That inasmuch as there is no definite constitution of the Fund and to obviate difficulties arising in the future the Council be authorised to take such steps as it thinks fit for the incorporation of the Fund by Royal Charter or otherwise.

He could have wished, he said, that the moving of that resolution had fallen into better hands than his own, and he could only suppose he had been chosen because for more than a quarter of a century his congregation had steadily, and though he was their clergyman he might say nobly, contributed to the Fund an average of £1,200 a year. The putting of that resolution into his hands was, he felt, an honourable tribute to a congregation which had done its part for the sick poor of London, and by its example had inspired others to do the same. As to the need for the resolution, although since the Fund was started they had worked very satisfactorily on the basis then laid down for this noble work, yet they had no definite constitution, and were in the strange position of being without the legal power to make by-laws. It was also an anomaly that people who personally contributed large sums, like Mr. George Herring, who gave some £12,000 annually to the Fund, had no vote. It was not intended to alter the position of the members who represented congregations, but to make arrangements for power to add others. It was desirable also that members of the Council should retire by rotation and be eligible for re-election. It seemed to him, inasmuch as this Fund had grown in public estimation, as was shown by the collections having increased from £27,000 in 1873 to £78,000 in 1905, that it ought now to be established on a basis that none could question, and that steps should be taken to incorporate it either by Royal Charter or otherwise, and so put it on a sound foundation for all time.

The Rev. W. H. Harwood, of Union Congregational Chapel, Islington, seconded the resolution. He said he imagined he had been asked to do so as representing another section of the community. He was sure it was in perfect harmony with the wishes of the congregations of the body with which he was connected, and that the proposal was made in order to safeguard the Fund so dear to them all. The desire to support that great organisation for the relief of the sick poor was common to all the great religious bodies of London, and they wished to make it more effective in the days to come. It was right that the Fund should now be put on a strictly legal basis, and he was sure there was no desire on the part of the Council or any of the constituents to stifle healthy discussion, and that it was possible to devise methods so that it would be as easy in the future as it had always been for the representatives of the subscribers to meet and talk over whatever was of interest or importance. Arrangements would be made to safeguard their honourable traditions. No work in the past had been more pleasantly and harmoniously done, and that accord would still be possible, even though they had rather more elaborate machinery.

The Chief Rabbi, Dr. Adler, said it afforded him extreme satisfaction to support the resolution, for the Hospital Sunday Fund had always been dear and sacred to his community, so that year by year the aggregate of the contributions had increased, the poorest congregations of the East End being glad to do their best in such a cause. The Council hoped to obtain a charter by means of which powers and privileges were conferred on a corporate body. Without such legal authority the acts of a society might be regarded as the acts of an individual. It was of the greatest importance that they should possess a constitution, and he hoped they might be conscious that greater stability was given to the work.

Sir Richard Biddulph Martin (Hon. Secretary) supported the resolution, remarking that it seemed a great many years ago since they had gathered in the London Tavern to discuss the possibility of originating the Hospital Sunday Fund for London, immediately obtaining the enthusiastic support of the then Lord Mayor, Sir Sidney Waterlow. It was certainly in the interest of the Fund that they should have a legal constitution; they had done very well in the past, and he was sure that in the future they would do still better.

A constituent, speaking from the body of the hall, suggested that the resolution should be so modified as to be a request to the Council to draft a scheme for submission to the constituents.

The Chairman said that was unnecessary, since the matter could not be carried to a conclusion without a further meeting, at which the matter would be submitted to the general body.

The resolution was then put and declared carried unanimously.

A vote of thanks to the Lord Mayor concluded the meeting.

